# Correlations between Different Heavy Metals in Diverse Body Fluids: Studies of Human Semen Quality

**DOI:** 10.1155/2012/420893

**Published:** 2012-01-24

**Authors:** Lidia Mínguez-Alarcón, Jaime Mendiola, Manuela Roca, José J. López-Espín, José J. Guillén, José M. Moreno, Stella Moreno-Grau, María J. Martínez-García, Nuria Vergara-Juárez, Belén Elvira-Rendueles, Antonio García-Sánchez, Jorge Ten, Rafael Bernabeu, Alberto M. Torres-Cantero

**Affiliations:** ^1^Public Health and Epidemiology Research Group, Division of Preventive Medicine and Public Health, School of Medicine, University of Murcia, Espinardo 30100, Murcia, Spain; ^2^Center of Operations Research, Miguel Hernández University, 03202 Elche, Spain; ^3^Department of Environmental and Chemical Engineering, Technical University of Cartagena, Cartagena 30202, Spain; ^4^Department of Reproductive Biology and Medicine, Instituto Bernabeu, Alicante 03016, Spain; ^5^Reproductive Medicine Chair, Miguel Hernández University-Instituto Bernabeu, Alicante 03016, Spain; ^6^Centro de Investigación Biomédica en Red de Epidemiología y Salud Pública (CIBERESP), Barcelona 08036, Spain

## Abstract

It has been hypothesized that exposure to heavy metals may impair male reproduction. To measure the effect produced by low doses of heavy metals on semen parameters, it is necessary to clarify in which body fluids those measurements must be performed. Sixty-one men attending infertility clinics participated in our study. Concentrations of lead, cadmium, and mercury were measured in whole blood, blood plasma, and seminal plasma using spectroanalytical and electrochemical methods. Semen analyses were performed according to World Health Organization criteria. For statistical analysis, Spearman's rank correlations, mean comparison tests, and discriminant analysis were calculated. Significant correlations between the measured concentrations of the three heavy metals in the same biological fluids were observed. However, no similar relationship was seen when comparing the concentrations in different body fluids of the same metal. According to our results and previous publications, seminal plasma might be the best body fluid for assessing impairment of human semen parameters.

## 1. Introduction

Over time there has been a significant decline of human fertility [1]. Like other European countries, Spain is since 1981 well below the 2.1 children needed to maintain replacement level [2]. Birth rate, have declined mainly due to changes in lifestyle and social mores and increased contraception [3].

These demographic transformations, as much as they are socially valued and desirable, have important clinical consequences. The fertility decline has resulted in a major delay in the average age of conception. The first pregnancy is postponed to ages at which women fecundity is decreased [4]. That may be one important reason why the medical reproductive units have taken on such a relevant role in developed countries. Between 2002 and 2004, more than 6% of Danish children were born through assisted reproduction techniques [1]. Consequently, social and medical considerations about infertility have become an important concern in recent years.

In parallel, it has been hypothesized that there is a worldwide decline in male semen quality [5–8], but it is clearly not uniform [9]. The decline in semen quality has been linked to environmental and work-related toxic exposures [10, 11]. For example, heavy metals may compromise male reproduction, as demonstrated by epidemiological and animal studies [12–22].

Our research interests are related to the measurement of the exposure to lead (Pb), cadmium (Cd), and mercury (Hg), and its relationship with human semen quality. The main results published on that issue are summarized in Table 1. Those studies were done using the World Health Organization (WHO) guidelines for semen analysis published in 1987 [23], 1992 [24], and 1999 [25]. However, in 2010, the WHO published new criteria for the assessment of semen samples [26], and all the sperm parameter cutoffs were lowered. The changes in the three main semen parameters through time (1987–2010) are summarized in Table 2.

### 1.1. Lead

There is considerable agreement that high or even moderate concentrations of lead cause fertility problems in humans. Fatima et al. showed that >40 *μ*g/dL of lead in blood produced a decline of sperm count (<20 × 10^6^ cells/mL). In addition, they observed lower motility (<50%) and morphology (<14%), with >35 *μ*g/dL in whole blood [12]. Telisman and colleagues showed significantly lower sperm density and motility with high blood lead concentrations (36.7 *μ*g/dL) [13]. High concentrations of lead seem to be clearly associated with sperm damage. 

However, there are conflicting results about the effect on semen quality at low lead exposures. Hernandez-Ochoa and colleagues found that low lead concentrations in seminal fluid (0.2 *μ*g/dL) were associated with impaired semen quality, 44% of motility, 32% of normal morphology, and 11 × 10^6^ cell/mL of sperm concentration [14]. In contrast, Mendiola et al. found a relationship between levels of lead ten times higher in the spermatic fluid (2.93 *μ*g/dL) and low motility, but no effect on morphology (>14%) or sperm concentration (>20 × 10^6^ cells/mL) [15]. Similarly, Hovatta et al. reported that lead concentrations in seminal plasma of 2.5 *μ*g/dL did not affect sperm concentration (96 × 10^6^ cells/mL) [16]. Moreover, Mendiola et al. found that lead concentrations of 9.75 *μ*g/dL measured in whole blood and 2.78 *μ*g/dL in blood plasma had no effect on morphology (>14%), motility (>50%), or sperm concentration (>20 × 10^6^ cells/mL) [15]. Meeker et al. also reported no effect on sperm concentration (42.7 × 10^6^ cells/mL) or motility (55%) with 1.5 *μ*g/dL of lead concentration in whole blood [17]. 

### 1.2. Cadmium

At high concentrations, cadmium could affect semen quality. According to Akinloye et al., men with high concentrations of cadmium in seminal plasma (65 *μ*g/dL) had 5.16 × 10^6^ cells/mL of sperm count and 36% of motile sperms [18]. 

As seen with lead, there is no agreement on the effect of low concentrations of cadmium on semen quality. Telišman et al. found that even low concentrations of cadmium <1 *μ*g/dL in whole blood were associated with head pathologic sperms [13]. Benoff and colleagues concluded that sperm concentration, motility, and morphology are affected even with low seminal plasma concentrations of cadmium (0.028 *μ*g/dL) [19]. Mendiola and colleagues also found that low concentrations of cadmium in seminal plasma (0.085 *μ*g/dL) were moderately associated with low sperm motility (<50%) but had no effect on morphology (>14%) or sperm concentration (>20 × 10^6^ cells/mL) [15]. Equally, Hovatta et al. showed no correlation between higher cadmium concentrations in seminal fluid (0.15 *μ*g/dL) and sperm concentration (96 × 10^6^ cells/mL) [16]. Chia and colleagues did not find an impairment of morphology (>50%) and motility (>50%) with low concentrations of cadmium in whole blood (0.095 *μ*g/dL) [20]. Similarly, Mendiola et al. showed that cadmium measured in whole blood (0.10 *μ*g/dL) and blood plasma (0.08 *μ*g/dL) did not impaired morphology (>14%), motility (>50%), or sperm concentration (>20 × 10^6^ cells/mL) [15]. Moreover, Meeker et al. reported no effect of low cadmium concentrations in whole blood (0.04 *μ*g/dL) on sperm density (42.7 × 10^6^ cells/mL) and motility (55%) [17]. 

### 1.3. Mercury

There is clear evidence that very high concentrations of mercury in the body will harm sperm. Choy et al. showed that high concentrations of total mercury (inorganic and organic) measured in whole blood (40.6 mmol/L) resulted in <50% of progressive motility, <14% of normal morphology, and <20 × 10^6^ cells/mL of sperm concentration [21]. 

However, Mendiola et al. did not find an alteration of motility (>50%), morphology (>14%), or sperm concentration (>20 × 10^6^ cells/mL) at low concentrations of total mercury in seminal plasma (1.18 *μ*g/dL). Besides, low concentrations measured in whole blood (1.99 *μ*g/dL) and blood plasma (0.6 *μ*g/dL) were not related to decreased morphology (>14%), motility (>50%), or sperm concentration (>20 × 10^6^ cells/mL) [15]. Rignell-Hydbom et al. found no association with sperm motility (54%) or concentration (48 × 10^6^ sperm cells/mL) at low concentrations of organic mercury in whole blood (0.225 *μ*g/dL) [22]. In addition, Meeker et al. reported that low mercury concentrations in whole blood (0.11 *μ*g/dL) did not affect motility (55%) and sperm concentration (42.7 × 10^6^ cells/mL) [17]. 

### 1.4. Justification of the Study

There are at least two problems in assessing whether low concentrations of heavy metals have an impact on human semen quality. First of all, there are just a few studies published on that issue so far. A second problem relates to the variables measured; that is the biological samples in which the concentrations of heavy metals are measured, and the parameters used to measure semen quality (motility, morphology, and sperm concentration). 

To measure the effect produced by low doses of a heavy metal in the reproductive organs, it is necessary to clarify where to perform those measurements. Concentrations of heavy metals may be measured in the whole blood, in blood plasma, and in seminal plasma. However, it is not clear whether measurements in one or another fluid are equivalent, nor to what extent there are correlations between the three measurements of these heavy metals in the different body fluids. 

The objectives of this study are (1) to examine whether there are correlations between the concentrations of heavy metals (lead, cadmium, and mercury) in the three body fluids (whole blood, blood plasma, and seminal plasma) and (2) to explore whether any one of the three measures relates better than the others with the semen quality parameters. 

## 2. Materials and Methods

### 2.1. Study Population, Design, and Semen Analysis

The study population, hormone, and semen analyses have been previously described elsewhere [27, 28]. Sixty-one men were participating in a study to explore the role of environmental toxins and lifestyles on male infertility. Briefly, the men of couples attending three infertility centers in southeastern Spain between 2005 and 2007 were classified on the basis of semen quality, following WHO criteria [25]. Subjects provided two semen samples and were requested to observe a 3- to 5-day abstinence period. The importance of the abstinence period was stressed on the interviews with the participants [27]. The average of the two samples was used in our statistical analysis. Semen parameters evaluated included ejaculate volume, sperm concentration, percentage of motile sperm, and percentage of normal forms following Kruger's strict criteria [25]. All patients were interviewed face-to-face by the same interviewer and completed a comprehensive occupational and lifestyle questionnaire [27]. This study was approved by the Institutional Review Board. Patients were included in the study after giving informed written consent. 

### 2.2. Measurements of Metals

A total of 181 biological samples were analyzed for Pb, Cd, and Hg, including 61 samples of seminal plasma, 61 of blood plasma, and 59 of whole blood, as two samples were lost during the study. Biological samples were dispensed into aliquots and frozen and stored at −40°C until analysis. Anodic stripping voltammetry (ASV) was used for measuring Pb and Cd concentrations. ASV was carried out using a voltamperometer with VA 663 stand and VA 608 controller (Metrohm 626, Herisau, Switzerland). The voltamperometric cell was equipped with a drop of mercury as the working electrode, an Ag/AgCl/KCl 3 M reference electrode, and a platinum auxiliary electrode. 

Determination of total Hg was carried out by thermal decomposition, amalgamation, and atomic absorption spectrophotometry, using a mercury analyzer with quartz sample boats (DMA-80 Direct Mercury Analyzer, Milestone, Shelton CT, USA). 

The highest grade purity reagents were employed in this procedure including nitric acid 65% and perchloric acid 70% (Suprapur, Merck, Darmstadt, Germany). The ultrapure water was purified with Millipore Simplicity 185 (Millipore GmbH, Molsheim, France) obtaining conductivity values of 0.054 *μ*S/cm. 

In order to prepare the working standard solutions, commercially available standard solutions for Pb 1 g/L and Cd 1 g/L (Tritisol, Merck, Darmstadt, Germany) and Hg 1 g/L (Certipur, Merck, Darmstadt, Germany) were used. The limits of detection (LOD) for the body's fluid metal levels were as follows: lead, 21 *μ*g/L; cadmium, 0.11 *μ*g/L, and mercury, 0.1 *μ*g/L. To guarantee the accuracy and precision of the applied technique regarding heavy metals, whole blood reference materials (Seronorm Trace Elements Whole Blood, SERO AS, Billingstad, Norway) were employed. 

### 2.3. Sample Preparation

Pb and Cd determinations were performed using 0.2 mL of the biological sample deposited inside of 25 mL borosilicate glass. Acid digestion was carried out by adding 2 mL of nitric acid and 2 mL of perchloric acid and evaporating it to dryness. Once the sample was dry and cooled down, 100 *μ*L of perchloric acid and 15 mL of double-distilled water were added, transferring the final volume into a voltamperometric cell. 

Biological samples were measured by ASV according to the following method [24]. Briefly, differential pulse (DP) with hanging mercury drop electrode (HMDE) was used, the voltage sweep was from −0.70 to +0.15 volts, and the peak voltage was located at −0.58 and −0.40 volts for Cd and Pb respectively. Deaeration, preconcentration, and resting time (without stirring) were 180, 120, and 40 seconds, respectively. Sensitivity was 0.05 nAmp/mm and 0.2 nAmp/mm for Cd and Pb, respectively. Standard addition method was applied to perform the current analyses, adding known values of a standard solution (2, 4, and 6 ng for Cd and 20, 40, and 60 ng for Pb) to obtain a calibration curve, then the values of the measurements were interpolated into that curve. Mercury determination was carried out following EPA method 7473 [29], and 0.2 mL of the biological sample was transferred directly into the quartz sample boats. To obtain a calibration curve, standard solutions of 5, 10, 20, 30, 100, 200, and 500 ng of Hg were employed. 

### 2.4. Statistical Analysis

The statistical analysis encompassed descriptive and inferential analyses. Basic, dispersion as well as frequency parameters were calculated for descriptive analyses. Statistical analyses were performed to explore possible patterns in the concentrations of heavy metals measured in blood serum, whole blood, and seminal plasma. Spearman's rank correlations and scatter plots were employed for comparison of variables. In the inferential analysis, the mean comparison tests and discriminant analysis were performed. All tests were two-tailed, and the level of statistical significance was set at 0.05. Statistical analysis was performed using SPSS 17.0 (SPSS Inc., Chicago, IL, USA). 

## 3. Results and Discussion

### 3.1. Results

Table 3 shows lead (Pb), cadmium (Cd), and mercury (Hg) concentrations in *μ*g/dL (mean, standard error, median, and interquartile range), in whole blood, blood plasma, and seminal plasma. 

**Figure 1:**
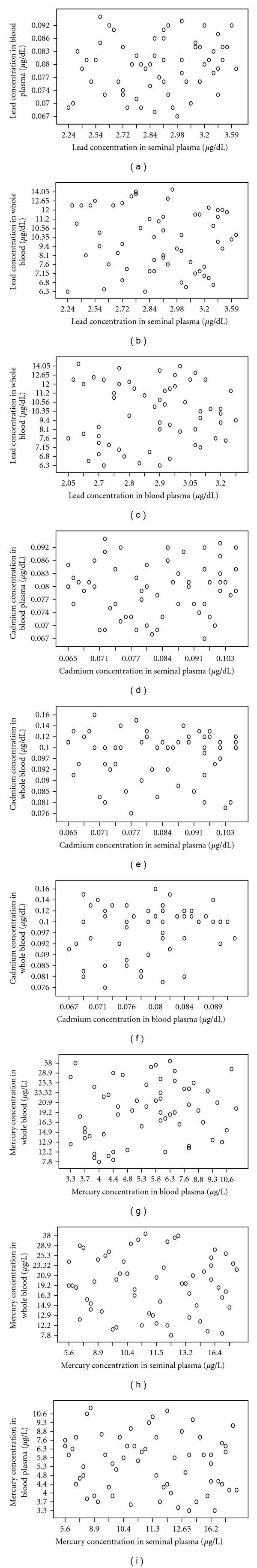
(a) Relation between lead concentrations in seminal plasma and blood plasma. (b) Relation between lead concentrations in seminal plasma and whole blood. (c) Relation between lead concentrations in blood plasma and whole blood. (d) Relation between cadmium concentrations in seminal plasma and blood plasma. (e) Relation between cadmium concentrations in seminal plasma and whole blood. (f) Relation between cadmium concentrations in blood plasma and whole blood. (g) Relation between mercury concentrations in blood plasma and whole blood. (h) Relation between mercury concentrations in seminal plasma and whole blood. (i) Relation between mercury concentrations in blood plasma and whole blood.

Figures 1(a)–1(i) show the scatter plots of the concentrations of the three metals in the three body fluids. As may be observed, men with low concentration of one heavy metal in a fluid can show low or high concentrations of the same metal in another fluid. There is a wide dispersion of data, and there are no associations between the measurements made of the metals in one fluid and the concentrations measured in the two other fluids. 

Table 4 presents the results of the Spearman's correlation between the concentrations of lead, cadmium, and mercury in whole blood, blood plasma, and seminal plasma. Although the correlation coefficients were above 0.5 for some determinations, no significant correlations were found between the concentrations of the same metal and the three biological fluids. The correlation between the concentration of lead in blood plasma and whole blood was 0.57 (*P* = 0.67), between cadmium in seminal plasma and whole blood was −0.50 (*P* = 0.72), and between mercury in seminal plasma and whole blood −0.34 (*P* = 0.80). 

**Figure 2:**
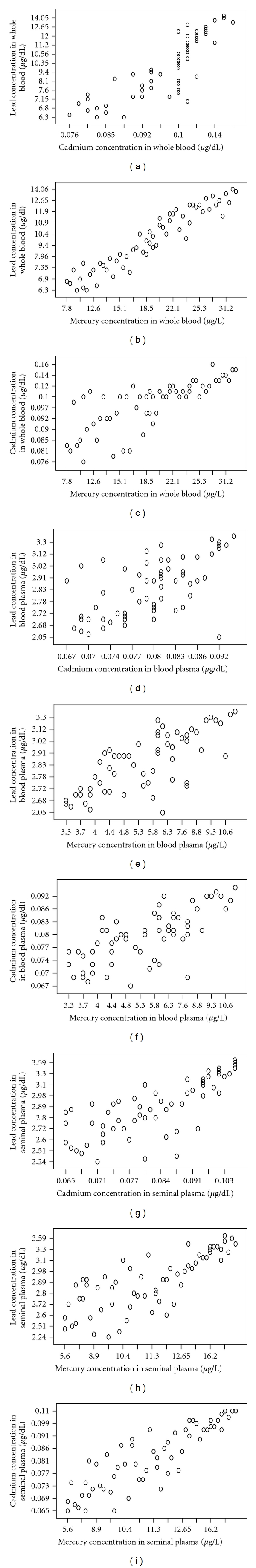
(a) Relation between lead and cadmium concentrations in whole blood. (b) Relation between lead and mercury concentrations in whole blood. (c) Relation between cadmium and mercury concentrations in whole blood. (d) Relation between lead and cadmium concentrations in blood plasma. (e) Relation between lead and mercury concentrations in blood plasma. (f) Relation between cadmium and mercury concentrations in blood plasma. (g) Relation between lead and cadmium concentrations in seminal plasma. (h) Relation between lead and mercury concentrations in seminal plasma. (i) Relation between cadmium and mercury concentrations seminal plasma.

Figures 2(a)–2(i) show the relationship between the concentrations of lead, cadmium, and mercury measured in each fluid. As may be observed, there is a linear relationship, since men with low concentration of a given metal in a biological fluid also had low concentration of the other two metals in the same fluid. And, reversely, men with high concentration of a given metal in a biological fluid also had high concentration of the other metals in the same body fluid. 

Spearman's correlation coefficients and scatter plots revealed a high correlation between the concentrations of the three metals in the same biological fluids.  Table 5 shows the correlation of the three heavy metals (Pb, Cd, and Hg) in the same biological fluid (whole blood, blood plasma, or seminal plasma). High and statistically significant correlations were observed between the three heavy metals for the same biological fluid. In seminal plasma, the correlation between cadmium and lead was 0.74 (*P*  value < 0.005) and between mercury and lead 0.76 (*P*  value < 0.005). 

To explore whether these correlations were determined by associations with other factors, exploratory scatter plots were generated between the concentrations of the three metals in the three biological fluids and possible confounding variables. Possible confounders were such as “occupation,” “tobacco smoke,” “exposure to toxics at work” or “using metals at work.” No patterns were observed. Hypothesis tests were used to detect significant differences in the mean concentrations of metals and the possible confounding factors used in the scatter plots. Not significant differences were found (data not shown). 

As a final alternative, metal concentrations were categorized in two, three, and four groups using the mean values, tertiles, and quartiles, respectively. Discriminant analysis was then used to detect whether any of the factors was related to the categories of the metal concentrations. To this end, different discriminant analysis evaluating the overall Wilks' lambda and the owners of each factor were produced, but none of them were satisfactory. 

### 3.2. Discussion

Using the Spearman's correlation coefficient and scatter plots revealed a high correlation between the measured concentrations of the 3 heavy metals in the same biological fluids. However, no similar relationship was observed when comparing the concentrations in different body fluids of the same metal. 

It would be reasonable to expect that subjects with high and low levels of exposure to any metal would show similar positions (low or high concentrations) in the measurements made in any body fluid. However, we found no correlation between the concentrations of any of the metal in the three biological samples analyzed (whole blood, blood plasma, and seminal plasma). 

Other authors, similarly, found no correlation between the concentrations of the same metal in different fluids [14, 19]. Benoff and colleagues found no correlation between cadmium concentrations in seminal plasma and blood plasma. Hernandez-Ochoa et al. also reported no correlation in blood lead concentrations between whole blood-plasma blood, whole blood-seminal plasma, or blood plasma-sperm in 68 Mexican men. 

There are some possible hypotheses for these phenomena. The three heavy metals are bound and transported by erythrocytes [30–32]. Given that metals are transported by red cells, unmeasured differences in the concentration of red cells in our study population may result in different concentration of the metals in the blood. However, this hypothesis cannot be tested, mainly due to information on red cell concentration was not collected. 

Surprisingly, the concentrations of Pb, Cd, and Hg were correlated in the same biological samples. Howatta et al. also found that the concentrations of cadmium and lead in seminal plasma were correlated [16]. We do not have a firm hypothesis of why that may happen. 

Correlations of the three heavy metals in the same body biological fluid may be due to an interaction between the different metals in the same compartment, so that the concentration of one metal determines the concentration of the others. We are not aware of lead, cadmium, or mercury modulate each other. However, it has been published that selenium produces the redistribution of Hg from plasma to erythrocytes at higher ratio [33] and the modification of hepatic zinc by cadmium [34]. Therefore, it could be that a given heavy metal might modulate proteins and/or enzymes in the cells and influence the concentration of other heavy metals. [35–37]. 

As to how to measure the effect produced by heavy metal concentrations on semen quality, it would be better to measure those metals in seminal plasma than in blood plasma or whole blood. Heavy metal concentrations in blood samples do not necessarily reflect the seminal plasma ones, since heavy metal concentrations reaching the seminal plasma could be quite different. 

Heavy metals have a strong capacity to induce oxidative stress in body cells by disintegration of the lipid membrane, and spermatozoa are quite sensible to oxidative stress [38, 39]. Thus, in principle, it would be more accurate to measure heavy metal concentrations in seminal plasma—than in other fluids—in order to determine sperm damage. Numerous antioxidants such as vitamin C, vitamin E, glutathione, coenzyme Q10, and some fruits may diminish the oxidative stress caused by heavy metals [28, 40, 41]. 

Furthermore, as it can be seen in Table 1, high concentrations of heavy metals can alter sperm morphology, motility, and concentration individually. However, an alteration of the three semen parameters can be observed with very low heavy metal concentrations only in seminal plasma, showing us that this body fluid might reflect better the sperm damage. 

Finally, our findings might be attributed to chance or bias. The sample of individuals included in the study was small and the lack of statistically significant correlations may be a consequence of that. Our findings are, however, consisting with those [14, 16, 19] of that have explored the same correlations leading us to believe that they cannot be attributed to random or systematic error. 

## 4. Conclusions

Our study suggests that there is no correlation between the concentrations of any of the metals in the three biological samples analyzed (whole blood, blood plasma, and seminal plasma) and there is a correlation between the concentrations of Pb, Cd, and Hg in the same biological samples. According to our results and previous publications, seminal plasma might be the best body fluid for assessing impairment of human semen parameters. 

## Figures and Tables

**Table 1:** Review of the measurement in the exposure to lead (Pb), cadmium (Cd), and mercury (Hg) and its relation with semen quality.

		Semen quality		
		Morphology	Motility	Sperm concentration
Lead	Whole blood	Fatima 2010 [12]:	Telišman 2000 [13]:	Fatima 2010 [12]:
		(i) C ≥ 35 *μ*g/dL	(i) C = 36.7 *μ*g/dL	(i) C ≥ 40 *μ*g/dL
		(ii) Mr ≤ 14%	(ii) Mt = *P* < 0.02	(ii) SpC ≤ 20 × 10^6^ cells/mL
		(iii) 1999 criteria	(iii) 1987 criteria	(iii) 1999 criteria
		Mendiola 2011 [15]:	Fatima 2010 [12]:	Telišman 2000 [13]:
		(i) C = 9.75 *μ*g/dL	(i) C ≥ 35 *μ*g/dL	(i) C = 36.7 *μ*g/dL
		(ii) Mr ≥ 14%	(ii) Mt ≤ 50%	(ii) SpC = *P* < 0.05
		(iii) 1999 criteria*	(iii) 1999 criteria	(iii) 1987 criteria
			Mendiola 2011 [15]:	Mendiola 2011 [15]:
			(i) C = 9.75 *μ*g/dL	(i) C = 9.75 *μ*g/dL
			(ii) Mt ≥ 50%	(ii) SpC ≥ 20 × 10^6^ cells/mL
			(iii) 1999 criteria*	(iii) 1999 criteria*
			Meeker 2008 [17]:	Meeker 2008 [17]:
			(i) C = 1.5 *μ*g/dL	(i) C = 1.5 *μ*g/dL
			(iii) Mt = 55%	(ii) SpC = 42.7 × 10^6^ cells/mL
			(iii) 1999 criteria*	(iii) 1999 criteria*
	Blood blasma	Mendiola 2011 [15]:	Mendiola 2011 [15]:	Mendiola 2011 [15]:
		(i) C = 2.88 *μ*g/dL	(i) C = 2.88 *μ*g/dL	(i) C = 2.88 *μ*g/dL
		(ii) Mr ≥ 14%	(ii) Mt ≥ 50%	(ii) SpC ≥ 20 × 10^6^ cells/mL
		(iii) 1999 criteria*	(iii) 1999 criteria	(iii) 1999 criteria
	Seminal blasma	Mendiola 2011 [15]:	Mendiola 2011 [15]:	Mendiola 2011 [15]:
		(i) C = 2.93 *μ*g/dL	(i) C = 2.93 *μ*g/dL	(i) C = 2.93 *μ*g/dL
		(ii) Mr ≥ 14%	(ii) Mt ≤ 50%	(ii) SpC ≥ 20 × 10^6^ cells/mL
		(iii) 1999 criteria*	(iii) 1999 criteria*	(iii) 1999 criteria*
		Hernández-Ochoa 2005 [14]:	Hernández-Ochoa 2005 [14]:	Hovatta 1998 [16]:
		(i) C = 0.2 *μ*g/dL	(i) C = 0.2 *μ*g/dL	(i) C = 2.5 *μ*g/dL
		(ii) Mr = 32%	(ii) Mt = 44%	(ii) SpC = 96 × 10^6^ cells/mL
		(iii) 1999 criteria	(iii) 1999 criteria	(iii) 1992 criteria
				Hernández-Ochoa 2005 [14]:
				(i) C = 0.2 *μ*g/dL
				(ii) SpC = 11 × 10^6^ cells/mL
				(iii) 1999 criteria

Cadmium	Whole blood	Telišman 2000 [13]:	Mendiola 2011 [15]:	Mendiola 2011 [15]:
		(i) C ≤ 1 *μ*g/dL	(i) C = 0.10 *μ*g/dL	(i) C = 0.10 *μ*g/dL
		(ii) Mr = *P* < 0.005	(ii) Mt ≥ 50%	(ii) SpC ≥ 20 × 10^6^ cells/mL
		(iii) 1987 criteria	(iii) 1999 criteria*	(iii) 1999 criteria*
		Mendiola 2011 [15]:	Chia 1994 [20]:	Meeker 2008 [17]:
		(i) C = 0.10 *μ*g/dL	(i) C = 0.095 *μ*g/dL	(i) C = 0.04 *μ*g/dL
		(ii) Mr ≥ 14%	(ii) Mt ≥ 50%	(ii) SpC = 42.7 × 10^6^ cells/mL
		(iii) 1999 criteria*	(iii) 1987 criteria	(iii) 1999 criteria
		Chia 1994 [20]:	Meeker 2008 [17]:	
		(i) C = 0.095 *μ*g/dL	(i) C = 0.04 *μ*g/dL	
		(ii) Mr ≥ 50%	(ii) Mt = 55%	
		(iii) 1987 criteria	(iii) 1999 criteria	
	Blood plasma	Mendiola 2011 [15]:	Mendiola 2011 [15]:	Mendiola 2011 [15]:
		(i) C = 0.08 *μ*g/dL	(i) C = 0.08 *μ*g/dL	(i) C = 0.08 *μ*g/dL
		(ii) Mr ≥ 14%	(ii) Mot ≥ 50%	(ii) SpC ≥ 20 × 10^6^ cells/mL
		(iii) 1999 criteria*	(iii) 1999 criteria*	(iii) 1999 criteria*
	Seminal plasma	Mendiola 2011 [15]:	Akinloye 2006 [18]:	Akinloye 2006 [18]:
		(i) C = 0.085 *μ*g/dL	(i) C = 65 *μ*g/dL	(i) C = 65 *μ*g/dL
		(ii) Mr ≥ 14%	(ii) Mt = 35.75%	(ii) SpC = 5.16 × 10^6^ cells/mL
		(iii) 1999 criteria*	(iii) 1999 criteria	(iii) 1999 criteria
		Bennof 2009 [19]:	Mendiola 2011 [15]:	Hovatta 1998 [16]:
		(i) C = 0.028 *μ*g/dL	(i) C = 0.085 *μ*g/dL	(i) C = 0.15 *μ*g/dL
		(ii) Mr = *P* < 0.05	(ii) Mt ≤ 50%	(ii) SpC = 96 × 10^6^ cells/mL
		(iii) 1992 criteria	(iii) 1999 criteria*	(iii) 1992 criteria
			Bennof 2009 [19]:	Mendiola 2011 [15]:
			(i) C = 0.028 *μ*g/dL	(i) C = 0.085 *μ*g/dL
			(ii) Mt = *P* < 0.05	(ii) SpC ≥ 20 × 10^6^ cells/mL
			(iii) 1992 criteria	(iii) 1999 criteria*
				Bennof 2009 [19]:
				(i) C = 0.028 *μ*g/dL
				(ii) SpC = *P* < 0.05
				(iii) 1992 criteria

Mercury	Whole blood	Choy 2002 [21]:	Choy 2002 [21]:	Choy 2002 [21]:
		(i) C = 40.6 mmol/L	(i) C = 40.6 mmol/L	(i) C = 40.6 mmol/L
		(ii) Mr ≤ 14%	(ii) Mr ≤ 50%	(ii) SpC ≤ 20 × 10^6^ cells/mL
		(iii) 1999 criteria	(iii) 1999 criteria	(iii) 1999 criteria
		Mendiola 2011 [15]:	Mendiola 2011 [15]:	Mendiola 2011 [15]:
		(i) C = 1.99 *μ*g/dL	(i) C = 1.99 *μ*g/dL	(i) C = 1.99 *μ*g/dL
		(ii) Mr ≥ 14%	(ii) Mt ≥ 50%	(ii) SpC ≥ 20 × 10^6^ cells/mL
		(iii) 1999 criteria*	(iii) 1999 criteria*	(iii) 1999 criteria*
			Rignell-Hydbom 2007 [22]:	Rignell-Hydbom 2007 [22]:
			(i) C = 0.225 *μ*g/dL	(i) C = 0.225 *μ*g/dL
			(ii) Mt = 54%	(ii) SpC = 48 × 10^6^ cells/mL
			(iii) 1999 criteria	(iii) 1999 criteria
			Meeker 2008 [17]:	Meeker 2008 [17]:
			(i) C = 0.11 *μ*g/dL	(i) C = 0.11 *μ*g/dL
			(ii) Mt = 55%	(ii) SpM = 42.7 × 10^6^ cells/mL
			(iii) 1999 criteria	(iii) 1999 criteria
	Blood plasma	Mendiola 2011 [15]:	Mendiola 2011 [15]:	Mendiola 2011 [15]:
		(i) C = 0.6 *μ*g/dL	(i) C = 0.6 *μ*g/dL	(i) C = 0.6 *μ*g/dL
		(ii) Mr ≥ 14%	(ii) Mt ≥ 50%	(ii) SpC ≥ 20 × 10^6^ cells/mL
		(iii) 1999 criteria*	(iii) 1999 criteria*	(iii) 1999 criteria*
	Seminal plasma	Mendiola 2011 [15]:	Mendiola 2011 [15]:	Mendiola 2011 [15]:
		(i) C = 1.18 *μ*g/dL	(i) C = 1.18 *μ*g/dL	(i) C = 1.18 *μ*g/dL
		(ii) Mr ≥ 14%	(ii) Mt ≥ 50%	(ii) SpC ≥ 20 × 10^6^ cells/mL
		(iii) 1999 criteria*	(iii) 1999 criteria*	(iii) 1999 criteria*

Note: This table shows author, publication year, concentration of metal in whole blood, blood plasma, and seminal plasma, their effect on semen quality parameters, and the WHO criteria used to classify the semen quality.

C: concentration of the metal, Mr: morphology, Mt: motility, SpC: sperm concentration.

*Mendiola et al. use Kruger's strict criteria (14% of normal forms) as a cutoff for sperm morphology [25].

**Table 2:** Changes in the three main semen parameters through time (1987–2010). A semen parameter was considered normal when the values were equal or above the presented figures [23–26].

	1987	1992	1999	2010
Sperm concentration (×10^6^ cells/mL)	20–200^1^	≥20	≥20	≥15
Motility (%)	≥60	≥50	≥50	≥40
Morphology (%)	≥50	≥30	≥14	≥4

^1^range.

**Table 3:** Heavy metal concentrations in seminal, blood plasma, and whole blood.

	Lead (*μ*g/dL)		Cadmium (*μ*g/dL)		Mercury (*μ*g/dL)	
	Mean (SE)	Median (IQR)	Mean (SE)	Median (IQR)	Mean (SE)	Median (IQR)
Blood plasma (*n* = 61)	2.88 (0.22)	2.90 (2.72–3.05)	0.08 (0.007)	0.08 (0.07–0.08)	0.6 (0.22)	0.58 (0.42–0.72)
Whole blood (*n* = 61)	9.75 (2.28)	10.10 (7.50–11.90)	0.10 (0.02)	0.10 (0.09–0.12)	1.99 (0.69)	1.96 (1.47–2.46)
Seminal plasma (*n* = 61)	2.93 (0.32)	2.90 (2.72–3.15)	0.08 (0.01)	0.08 (0.07–0.09)	1.18 (0.35)	1.13 (0.92–1.49)

SE: standard Error, IQR: interquartile range.

**Table 4:** Spearman's correlation coefficients between metal concentrations in seminal and blood plasma, and whole blood.

		Blood plasma		Whole blood	
		*R*	*P* value	*R*	*P* value
Lead	Blood plasma			0.57	0.67
	Seminal plasma	0.13	0.32	−0.08	0.55
Cadmium	Blood plasma			0.14	0.30
	Seminal plasma	0.12	0.36	−0.50	0.72
Mercury	Blood plasma			0.17	0.19
	Seminal plasma	−0.13	0.34	−0.34	0.80

**Table 5:** Spermean's correlation coefficients between seminal plasma, blood plasma, and whole blood, with metal concentrations.

		Cadmium		Mercury	
		*R*	*P* value	*R*	*P* value
Seminal plasma	Lead	0.740	0.001	0.760	0.001
	Cadmium			0.870	0.001
Blood plasma	Lead	0.550	0.001	0.750	0.001
	Cadmium			0.700	0.001
Whole blood	Lead	0.850	0.001	0.950	0.001
	Cadmium			0.792	0.001

## References

[1] Skakkebæk NE, Jørgensen N, Main KM (2006). Is human fecundity declining?. *International Journal of Andrology*.

[2] INE http://www.ine.es/en/inebmenu/mnu_analisis_en.htm.

[3] Mills M, Rindfuss RR, McDonald P, Te Velde E (2011). Why do people postpone parenthood? Reasons and social policy incentives. *Human Reproduction Update*.

[4] Dunson DB, Baird DD, Colombo B (2004). Increased infertility with age in men and women. *Obstetrics and Gynecology*.

[5] Carlsen E, Giwercman A, Keiding N, Skakkebaek NE (1992). Evidence for decreasing quality of semen during past 50 years. *British Medical Journal*.

[6] Irvine S, Cawood E, Richardson D, MacDonald E, Aitken J (1996). Evidence of deteriorating semen quality in the United Kingdom: birth cohort study in 577 men in Scotland over 11 years. *British Medical Journal*.

[7] Spanò M, Toft G, Hagmar L (2005). Exposure to PCB and p,p′-DDE in European and inuit populations: impact on human sperm chromatin integrity. *Human Reproduction*.

[8] Hauser R (2006). The environment and male fertility: recent research on emerging chemicals and semen quality. *Seminars in Reproductive Medicine*.

[9] Swan SH, Brazil C, Drobnis EZ (2003). Geographic differences in semen quality of fertile U.S. males. *Environmental Health Perspectives*.

[10] Indulski JA, Sitarek K (1997). Environmental factors which impair male fertility. *Medycyna Pracy*.

[11] Rubes J, Selevan SG, Evenson DP (2005). Episodic air pollution is associated with increased DNA fragmentation in human sperm without other changes in semen quality. *Human Reproduction*.

[12] Fatima P, Debnath BC, Hossain MM (2010). Relationship of blood and semen lead level with semen parameter. *Mymensingh Medical Journal*.

[13] Tališman S, Cvitković P, Jurasović J, Pizent A, Gavella M, Ročić B (2000). Semen quality and reproductive endocrine function in relation to biomarkers of lead, cadmium, zinc, and copper in men. *Environmental Health Perspectives*.

[14] Hernández-Ochoa I, García-Vargas G, López-Carrillo L (2005). Low lead environmental exposure alters semen quality and sperm chromatin condensation in northern Mexico. *Reproductive Toxicology*.

[15] Mendiola J, Moreno JM, Roca M (2011). Relationships between heavy metal concentrations in three different body fluids and male reproductive parameters: a pilot study. *Environmental Health*.

[16] Hovatta O, Venäläinen ER, Kuusimäki L, Heikkilä J, Hirvi T, Reima I (1998). Aluminium, lead and cadmium concentrations in seminal plasma and spermatozoa, and semen quality in Finnish men. *Human Reproduction*.

[17] Meeker JD, Rossano MG, Protas B (2008). Cadmium, lead, and other metals in relation to semen quality: human evidence for molybdenum as a male reproductive toxicant. *Environmental Health Perspectives*.

[18] Akinloye O, Arowojolu AO, Shittu OB, Anetor JI (2006). Cadmium toxicity: a possible cause of male infertility in Nigeria. *Reproductive Biology*.

[19] Benoff S, Hauser R, Marmar JL, Hurley IR, Napolitano B, Centola GM (2009). Cadmium concentrations in blood and seminal plasma: correlations with sperm number and motility in three male populations (infertility patients, artificial insemination donors, and unselected volunteers). *Molecular Medicine*.

[20] Chia SE, Xu B, Ong CN, Tsakok FMH, Lee ST (1994). Effect of cadmium and cigarette smoking on human semen quality. *International Journal of Fertility and Menopausal Studies*.

[21] Choy CMY, Lam CWK, Cheung LTF, Briton-Jones CM, Cheung LP, Haines CJ (2002). Infertility, blood mercury concentrations and dietary seafood consumption: a case-control study. *An International Journal of Obstetrics and Gynaecology*.

[22] Rignell-Hydbom A, Axmon A, Lundh T, Jönsson BA, Tiido T, Spano M (2007). Dietary exposure to methyl mercury and PCB and the associations with semen parameters among Swedish fishermen. *Environmental Health*.

[23] World Health Organization (1987). *WHO Laboratory Manual for the Examination of Human Semen and Semen-Cervical Mucus Interaction*.

[24] World Health Organization (1992). *WHO Laboratory Manual for the Examination of Human Semen and Human Sperm-Cervical Mucus Interaction*.

[25] World Health Organization (1999). *WHO Laboratory Manual for the Examination of Human Semen and Semen-Cervical Mucus Interaction*.

[26] World Health Organization (2010). *WHO Laboratory Manual for the Examination of Human Semen and Semen-Cervical Mucus Interaction*.

[27] Mendiola J, Torres-Cantero AM, Moreno-Grau JM (2008). Exposure to environmental toxins in males seeking infertility treatment: a case-controlled study. *Reproductive BioMedicine Online*.

[28] Mendiola J, Torres-Cantero AM, Moreno-Grau JM (2009). Food intake and its relationship with semen quality: a case-control study. *Fertility and Sterility*.

[29] EPA method 7473 Mercury in solids and solutions by termal decomposition, amalgamation, and atomic absorption spectrophotometry. http://www.epa.gov/sam/pdfs/EPA-7473.pdf.

[30] ATSDR: agency for toxic substances & disease registry Public health statement for cadmium. http://www.atsdr.cdc.gov/.

[31] ATSDR: agency for toxic substances & disease registry Toxicologycal profile for lead. http://www.atsdr.cdc.gov/.

[32] ATSDR: agency for toxic substances & disease registry Toxicologycal profile for mercury. http://www.atsdr.cdc.gov/.

[33] Orct T, Lazarus M, Jurasović J, Blanuša M, Piasek M, Kostial K (2009). Influence of selenium dose on mercury distribution and retention in suckling rats. *Journal of Applied Toxicology*.

[34] Braga MM, Dick T, Oliveira DL (2012). Cd modifies hepatic Zn deposition and modulates *δ*-ALA-D activity and MT levels by distinct mechanisms. *Journal of Applied Toxicology*.

[35] Coddou C, Lorca RA, Acuña-Castillo C, Grauso M, Rassendren F, Huidobro-Toro JP (2005). Heavy metals modulate the activity of the purinergic P2X4 receptor. *Toxicology and Applied Pharmacology*.

[36] Borges VC, Santos FW, Rocha JBT, Nogueira CW (2007). Heavy metals modulate glutamatergic system in human platelets. *Neurochemical Research*.

[37] Korashy HM, El-Kadi AOS (2008). Modulation of TCDD-mediated induction of cytochrome P450 1A1 by mercury, lead, and copper in human HepG2 cell line. *Toxicology in Vitro*.

[38] Ercal N, Gurer-Orhan H, Aykin-Burns N (2001). Toxic metals and oxidative stress part I: mechanisms involved in metal-induced oxidative damage. *Current Topics in Medicinal Chemistry*.

[39] Grotto D, Valentini J, Fillion M (2010). Mercury exposure and oxidative stress in communities of the Brazilian Amazon. *Science of the Total Environment*.

[40] Sheweita SA, Tilmisany AM, Al-Sawaf H (2005). Mechanisms of male infertility: role of antioxidants. *Current Drug Metabolism*.

[41] Tito A, Carola A, Bimonte M (2011). A tomato stem cell extract, containing antioxidant compounds and metal chelating factors, protects skin cells from heavy metal-induced damages. *International Journal Cosmetic Science*.

